# Airport Cement Concrete with Ceramic Dust of Increased Thermal Resistance

**DOI:** 10.3390/ma15103673

**Published:** 2022-05-20

**Authors:** Małgorzata Linek

**Affiliations:** Faculty of Civil Engineering and Architecture, Kielce University of Technology, Tysiąclecia Państwa Polskiego Street 7, 25-314 Kielce, Poland; linekm@tu.kielce.pl

**Keywords:** airport pavements, cement concrete pavements, durability of the concrete surfaces

## Abstract

The impact of aircraft on airport pavements is varied and closely related to their operational durability. The article presents the impact of the annealing process related to the forced impact of airplanes on airport pavements. The composition of cement concrete with ceramic dust, which is characterized by increased thermal resistance, has been proposed. Two research cycles were programmed, differentiated by the annealing scheme and the way in which the temperature influences the annealing time. Samples stored at a temperature of 20 ± 2 °C were subjected to testing. The tests were carried out for two diagrams: A and B. The first—diagram A—included the continuous impact of the flue gas stream on the samples for a period of 350 min with a test step every 25 min. For the second—diagram B—the samples were alternately heated (1 min) and cooled (15 min). The influence of the proposed pavement mix on changes in the internal structure of cement concrete and the increase in its resistance to high temperatures was determined. In the microstructure of the CC-1 concrete matrix, it was found that there were plate-granular portlandite crystals up to 10 µm in size and ettringite crystals with a length of 8 µm. In the CC-2 concrete, the ettringite crystals were less numerous and had a length of up to 5 µm, there were also continuous contact zones between the aggregate grains and the cement matrix (diagrams A). The alternating annealing/cooling (diagram B) resulted in the ettringite crystals in the CC-1 matrix being up to 10 µm long, and in the CC-2 concrete up to 7 µm long. The contact zone between the aggregate grain and the matrix in CC-2 concrete was continuous, and the microcracks in CC-1 concrete were up to 8 nm. Regardless of the heating diagram, in the surface zone, there were larger microcracks in the CC-1 concrete than in the CC-2 concrete. For diagram A they were 14 µm and 4 µm and for diagram B they were 35 µm and 5 µm, respectively. It was found that concrete with ceramic dust is characterized by a lower and more stable temperature increase. In scheme A, the average temperature increase on the heated surface ranged from 46 °C to 79.5 °C for CC-1 concrete, and from 33.3 °C to 61.3 °C for CC-2 concrete. However, in scheme B, the temperature after 350 heating cycles for CC-1 concrete increased to 129.8 °C, and for CC-2 concrete to 116.6 °C. After the cooling period, the temperature of CC-1 and CC-2 concrete was comparable and amounted to 76.4 C and 76.3 °C, respectively. CC-2 concrete heats to lower values, and favorable changes in internal structure translate into higher strength and durability (after 350 heating cycles according to scheme A, the strength of CC-1 concrete was 67.1 MPa and of CC-2 concrete 83.9 MPa, while in scheme B, respectively, 55.4 MPa for CC-1 and 75 MPa for CC-2).

## 1. Introduction

Airfield pavements have a specific system of structural layers designed for the traffic of aircraft and wheeled vehicles of airport service [1]. During the intensive development of air transport, the quality of airfield concrete pavements determines the opportunity for safe flight operations. The operational durability of the structure is the result of the selection of concrete mix components, distinguished by the required parameters, as well as differentiation of aggregate composition in terms of properly specified conditions of aggressive influence of the environment on pavements. The factor which determines the pavement’s durability is the quality of works conducted at all stages of the construction process–starting from the technology of incorporation compaction and curing of the mixture within the construction. An indispensable element of operational capacity within the assumed period of service life is the accepted maintenance policy and use of the pavement. The essence of loads generated on airfield pavements was discussed, among others, in the following works [2,3,4,5,6,7,8]. The opportunity of using cement concrete for transportation pavements with the aim of extending the period of non-failure operation was presented in the following presentations [9,10,11,12,13,14]. The author of the work [9,13,14] points out that when properly designed and built out of durable materials, concrete pavements can provide many decades of service with little maintenance. Related guidelines have been formulated, including the selection and modeling of the concrete mix composition influencing the durability of the structure. The paper [10] presents the results of research on the possibility of using recycled aggregates. A modified design method was established taking into account the optimal water content and material structure with a limited content of fine aggregates. The work [12] presents recommendations related to the rational selection of cement concrete composition in terms of increasing its durability and taking into account the degree of frost resistance of concrete. A method of selecting rational material compositions of the cement-concrete pavement depending on the conditions of its use was proposed. The paper [14] presents guidelines for the use of permanent rigid pavements in Europe. Various designs and structures of the pavement, the generated loads and concrete parameters, the occurring damages and their causes as well as the scope of further maintenance of the pavement are presented. The following authors noticed the necessity of introducing quantitative corrections in the case of material composition as a factor that provides a suitable quality of concrete [15,16,17,18,19,20,21,22,23,24,25,26,27,28,29,30,31]. The selection of component materials for the concrete mix that meets the requirements has been discussed in the works [16,21,22,25,28,31]. An important factor shaping the parameters of concrete is the type and properties of the aggregate. They were discussed, among others in [15] in terms of alkaline reactivity, in [20] in relation to aggregate damage, and in [18] in terms of the assessment of the impact on the existing mechanisms of pavement damage. The role of cement in a concrete mix was discussed in [17,24,26] in relation to standard requirements, in [19] in terms of cooperation with admixtures, and in [23], taking into account the compatibility of admixtures and additives. The application of the modification of the concrete composition is also widely presented in [27], and the identification and evaluation methods are presented in [29,30]. The durability of airfield pavements has been identified with the opportunity of the non-failure structure operation and taking into account the influence of diversified environmental conditions. Airfield pavement loading should be considered as the combination of interdependent factors distinguished by the diversified nature, range and method, and intensity of influence. Aircraft loads generated on the airfield pavements which could be of static, dynamic, or thermal nature should be considered as well. Static loads are identified as the maximum vertical impact of wheels of the undercarriage leg of the considered plane [3]. Aircraft generate dynamic loads on the pavements. Specificity of loads in the case of airfield structures is identified also with the particular stages of aircraft traffic and variable loading nature which directly results from aerodynamic rules of aircraft movement on the pavement (i.e., increasing or decreasing of the mass acting on the pavement together with the change of movement speed). The scope of environmental impact on airport systems depends on real conditions, results from service life, and the applied methods and procedures of maintenance conducted on the pavements in order to provide ground traffic safety [4,8,32]. Concrete pavements during the operations are also exposed to constant and adverse effects of external environmental conditions on a daily and annual temperature variations basis. These issues were discussed in the following works [33,34,35,36,37,38]. The author of the work [33] presented the factors that shape the need for proper concrete care and guarantee the correct course of the hydration process during maturation. The influence of curing on obtaining the expected concrete properties was determined. The essence of the construction of the internal microstructure of concrete is discussed in detail in [34], and methods for the evaluation of hardened concrete in terms of the porosity characteristics are also presented. The paper [35] presents the results of the influence of the type of surface treatment on the freeze-thaw durability of airport pavement concrete under the coupled effect of freeze-thaw cycles, ultraviolet radiation, abrasion, and flame erosion. On the basis of the obtained results, it was found that the highest efficiency occurs for the following materials: silane, modified polyurea, AH material, and epoxy resin. Environmental and thermal loads cause the stress of concrete slabs in pavements. Examples of works in this field [36,37,38] indicate the need for an individual approach to the dimensioning of concrete slabs, with particular emphasis on local conditions. As far as aircraft operation is concerned, it is also necessary to consider the influence of utilities (e.g., jet fuel, diesel fuel, hydraulic fluids, lubricating oils, aviation grease), which, in case of emergency situations, could have an impact on cement concrete of the carriageway [39,40,41]. The particular type of the destructive impact of aircraft are areas of imposed thermal loads appearing recurrently. Loads of this kind occur due to hot exhaust gas emissions from the nozzles of aircraft taking off. The degree of exposure of pavements to these destructive factors depends on geometrical parameters of exhaust nozzle and temperature and flow rate of the gas stream; spatial parameters of engine position with respect to slab surface, and structural elements of the power transmission system; angle of inclination of the motor axis, under which hot exhaust gas hits the pavement; the time of gas influence on the pavement; coexisting weather conditions; the condition and type of pavement and the pavement material properties.

**Figure 1:**
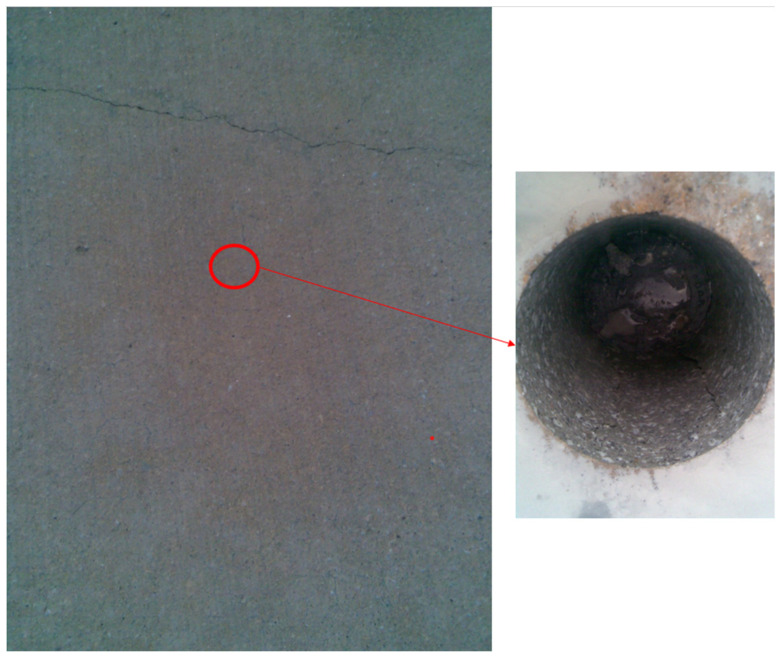
The operated airport rigid pavement with a visible color change within the area of so-called stream core and the place from which the test core was taken.

**Figure 2:**
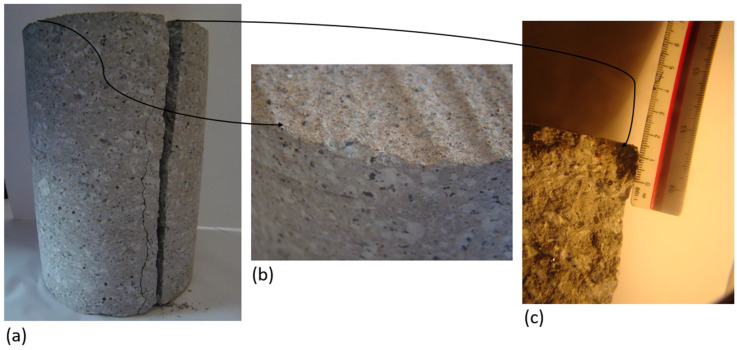
A test core taken from the used concrete airport pavement: (**a**) test core, (**b**) surface layer of the sample, and (**c**) cross-section of subsurface layer (sample diameter: 150 mm, sample height: 300 mm).

The work [4] specifies the details related to the characteristics of the hot flue gas stream that spreads on the airport pavement. The point of contact of the gas stream and the area of its propagation on the pavement as the “stream core” were determined. For this core, geometrical parameters were given (width of the order of 5–10 m and length of 70–90 m) and they were made dependent on the design parameters of the aircraft. The factors influencing the intensity of the temperature impact in the range of the engine axis and its extension as well as the inclination angle of the generated exhaust gases in relation to the road surface were determined. In the field tests, the temperatures to which the pavement may heat up as a result of this type of impact were determined, and it was stated in [42] that they may exceed the value of 200 °C. In the actual section of the airport pavement, a change in the color of the pavement (Figure 1) from gray to dark-gray-yellow was diagnosed, which, according to the literature [2], may indicate the influence of temperatures above 600 °C. The reaction of concrete in the surface layer is varied and depends on external factors. Based on the results analyzed in [2], it was determined that the impact zone extends into the road layer to a depth of 0.05 m. This is also confirmed by the observations made on the section of the pavement in question. On the basis of field studies, it was found that the scope of the impact deep into the near-surface zone of the road layer is 0.015 m (Figure 2).

According to [43], the gas stream in convergent nozzles at the pressure of approx. 200 kPa and exhaust temperatures without afterburning of 900 K, are distinguished by the gas flow rate of 550 m/s, while using the afterburning, the gas flow rate is approx. 700 m/s. In the case of the most recent engines, the temperature with afterburning is up to 2000 K, and a gas flow rate of approx. 1000 m/s. The stream range is distinguished by: aerodynamic, physical, and geometrical parameters, flow rate and temperature of exhaust gas, angle of inclination of the gas stream with respect to the pavement, and the width of the stream range. The depth of the stream range during the engine operation while the plane takes off exceeds 200 m, and the width amounts to 40–80 m. The hot exhaust gas stream, after leaving the engine nozzle, while heating the airfield pavement, spreads as an ellipsoidal shape creating the stream area which has been discussed in detail in the following works [2,43,44,45].

To an increased extent, the destructive effects of aircraft are recorded at military airports. In [46] the authors note the risk of regular hydrocarbon exposure, extreme thermal shocks, and repetitive loads. The paper [47] presents the results of research on the possibility of using hybrid fibers to protect the pavement against spalling damage. These fibers were used to increase the mechanical and thermal properties of concrete. The authors of the work [47] showed that the reinforcement of concrete with polyvinyl alcohol, steel, and hybrid fibers does not protect against the loss of compressive strength. The reduction of the examined feature was strictly dependent on the type of fibers used. It was also noted that the hybridization of concrete increases the thermal conductivity and bending strength values (by 22% and 47%, respectively) and reduces the appearance of spalling. However, in the work of [48], the strength parameters of concrete were analyzed in conditions simulated at the airport. These studies included the effect of operating media used in airplanes and elevated temperature as a function of the effect on cement materials. The authors [48] showed that the tested epoxy mortar is resistant to saponification, while numerous thermal cracks appeared on the Portland mortar, but epoxy and geopolymer did not experience any visual thermal cracks under the same conditions.

An important environmental aspect is a possibility of reusing materials in the composition of concrete mixtures. In [49], the author emphasized the need for recycling and discussed the situation in the world in this regard. Successful application of RA in the production of self-compacting and roller-compacted concrete is discussed. The paper [50] presents a set of case studies on the use of RA (recycled aggregates) in structural and non-structural elements in the construction of road pavements and buildings. The authors in [51] discussed the possibilities of using RAC for use in road pavement elements. The road was put into service in 1938 and after 82 years of use, the surface was completely recycled. This involved crushing the existing concrete and reusing it in new pavement layers. Based on the experimental studies presented in [52], it was shown that in the construction of concrete, it is recommended to limit the use of demolished coarse-grained aggregate derived from recycling and fly ash.

The specific nature of pavement operation, the necessity of using the structure in diversified environmental conditions, and providing high criteria of traffic safety are determined by the necessity of providing relevant concrete quality of the pavement. Particular attention should be paid to the opportunity of using modern technological solutions. This paper presents the extended scope of experimental tests on the effect of the heat flux generated on the engine dynamometer. In the comparative analysis, the influence of the pavement mix used on the thermal resistance of concrete and its internal structure was determined.

## 2. Concrete Durability Criteria in Case of Airfield Structure

The durability of airfield pavements is understood as the opportunity of failure-free operation within the assumed operation period, and, taking into consideration the influence of diversified environmental conditions, is specified as several coexisting and interdependent factors. The issue of concrete structure’s durability, in terms of the multi-criteria aspects, have been discussed, among other issues, in the following works [34,53,54,55].

The applicable standard [56] includes the recommendations concerning the properties of concrete designed for airfield pavements. In terms of structure durability, at the technological stage, component material requirements, defined air contents, and concrete mix consistency class were specified, and minimum class requirements and the required concrete parameters were defined. Issues concerning water absorption range and frost resistance degree providing structure durability were taken into consideration. Provisions of the following standards [57,58] were referred to the requirements included in [59], according to which provision of concrete durability is equivalent to complying, among others, with the requirements regarding the selection of component materials of specified parameters, maintaining minimum cement and air content, and maximum w/c indicator in the concrete mix, obtaining minimum concrete compressive strength, and provision of protection against drying out after concrete molding.

General requirements for airport pavements are defined in the paper [1]. According to the requirements of the standard [56], cement concrete intended for the construction of airfield pavements should contain Portland cement of clean clinker type, which minimum class is CEM I 32.5. Cement should be of low-alkali type (contain below 0.6% Na_2_O). Cement content in the concrete mix is diversified and closely dependent on the parameters of the designed concrete. Airfield pavement concrete should be resistant to environmental impacts, such as frost corrosion (XF4), corrosion caused by carbonatation (XC4), corrosion caused by chlorides (XD3), the abrasion impact (XM3), and class (XA2) [24,28]. The requirements for the concrete composition are included in Table 1.

The concrete mix includes water in the amount dependent on the assumed w/c ratio. The maximum value of w/c ratio according to standard requirements [56] is 0.4. The most significant component of the concrete mix is aggregate. Aggregate content in the mix is 70–75%. The type of the aggregate used is of primary importance in shaping the parameters of the hardened concrete. The aggregate used in the case of airfield pavements, according to [56] should comply with the requirements included in Table 2. 

Grain-size distribution curves of the designed mixes should be in between limit curves (lower and upper) which indicate the range of good grain size distribution, in accordance with [56]. The following evaluation criteria for stone materials were accepted:

- in the case of coarse aggregate their geometrical characteristics (aggregate dimensions, grain-size distribution according to [60], dust content and dust quality according to [60], physical and mechanical (crushing resistance according to [61], polishing resistance according to [62], abrasion resistance [63] grains density and absorbability according to [64], frost resistance according to [65]), a chemical composition according to the requirements of [66] and compared with the requirements of [56];
- in the case of fine aggregate, the geometrical, physical, and mechanical characteristics were defined according to the requirements of [66] and they were compared with the requirements of [56].
- In the case of the remaining types of material included in the mix composition, the requirements of [56,57,59] were taken into consideration with the following criteria:
- in the case of Portland cement, compliance with the requirements of [67];
- in the case of the water, compliance with the requirements of [68]. The used water should not have any smells (e.g., putrid). Water pH value should be ≥4, H_2_S content ≤ 20 mg/dm^3^, sulfates content ≤ 600 mg/dm^3^, and carbohydrate content ≤ 500 mg/dm^3^;
- in the case of the admixture, compliance with the requirements of [69].
- in the case of the designed concrete mixes, their physical properties were determined according to the requirements of specialist standards and [70], as shown in Table 3.

In the scope of experimental works that enable the assessment of concrete parameters, it is required to mark the possibility of using the designed concrete for specific construction purposes. The assessment of the suitability of concrete for the construction of airport pavements means, among others, the need to determine selected mechanical and physical parameters of concrete. These parameters, in accordance with [56], should be determined after the standard, i.e., a 28-day period of maintenance, in accordance with the detailed provisions. As part of the assessment, the following parameters should be determined:

- Compressive strength in accordance with the guidelines [74]. The tested feature, depending on the designed concrete class and the size of the test bodies (cylindrical samples 150 mm × 300 mm or cubic samples 150 mm × 150 mm × 150 mm), should be for the C30/37 class, higher than 30 MPa or 37 MPa, respectively; for the C35/45 class, higher than 35 MPa or 45 MPa; for the C40/50 class, higher than 40 MPa or 50 MPa, and; for the C45/55 class, higher than 45 MPa or 55 MPa;
- Bending strength in accordance with the guidelines of [75]. The tested property should be for the C30/37 class higher than 5.0 MPa; for the C35/45 class higher than 5.5 MPa; for the C40/50 class higher than 5.6 MPa, and; for the C45/55 class higher than 5.7 MPa;
- Tensile strength during splitting in accordance with the guidelines of [76]. The tested property should be for the C30/37 class higher than 3.3 MPa, for the C35/45 class higher than 3.6 MPa, for the C40/50 class higher than 3.7 MPa, and for the C45/55 class higher than 3.8 MPa;
- Absorbability of concrete in water and de-icers according to [56]. The tested property should be for the C30/37 class not exceeding 5.0%, for the C35/45 class not exceeding 4.9%, for the C40/50 class not exceeding 4.9%, and for the C45/55 class not exceeding 4.8%;
- Frost resistance of concrete according to [56], which in the case of airfield pavements should be F200 (after 200 freezing and de-freezing cycles, loss of weight and loss of resistance should not exceed accordingly 5.0% and 20% for class C30/37, 4.9% and 19% for class C35/45, 4.8% and 18% for class C40/50 and 4.7% and 17% for class C45/55);
- Resistance to surface flaking of concrete, according to [56], should not exceed 0.01 kg/m^2^;
- No phenomenon of permeability of water through concrete is specified according to [77].

Obtaining durable concrete composite, apart from the aforementioned factors, is referred to the technological process concerning proper concrete incorporation, compaction, and curing, and the maintenance process being conducted on time during the operation. The discussed determining factors of concrete durability presented in the following works [2,24,34,78,79,80], refer to the issue of cement concrete microstructure and changes within. A crucial role was attributed to the aggregate amount and type and the shape of grains, their porosity, and the component’s reactivity was considered particularly important. The cement matrix composition and the structure of contact areas with aggregate grains, micro-cracks occurring within the area of matrix, or contact areas and porosity characteristics are reflected in permeability of aggressive utilities and the extent of capillary action explicitly influencing concrete structure durability.

## 3. Materials and Methods

### 3.1. Materials: Aggregate and Cement

The reactive and physical and mechanical properties of the aggregate used has a significant impact on the amount of rheological deformation of concrete. The tests performed and the results presented in [81] showed that the materials are not reactive in terms of alkali, see Table 4. In the case of the selected types of coarse aggregate, the following were determined: volume density (ρa) according to [82]—2.65 Mg/m^3^, absorbability (W) according to [63]—0.6%, abrasion resistance (M_DE_) according to [67]—M_D10,_ and crushing resistance (LA) according to [61]—LA_35_.

**Figure 3:**
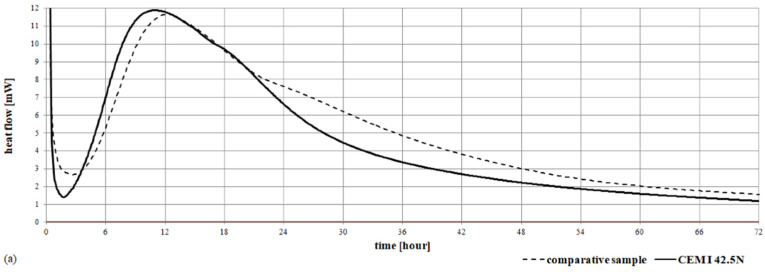

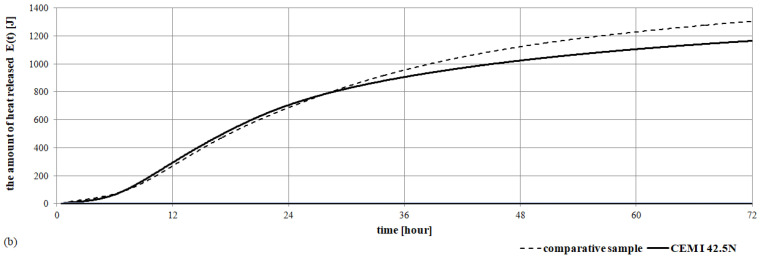

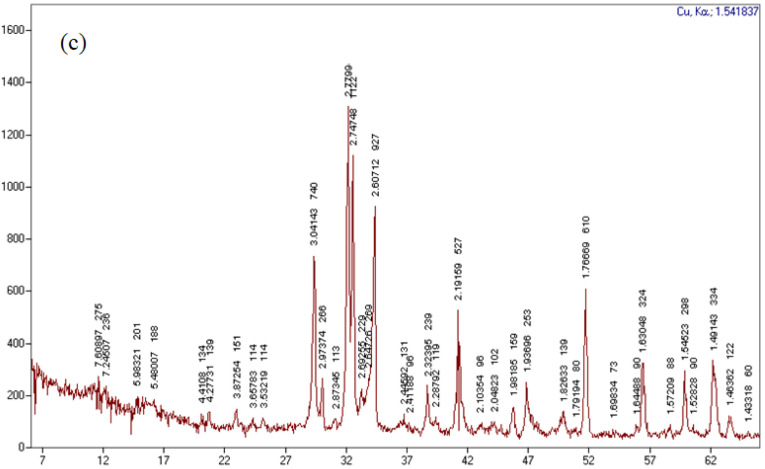
Cement characteristics: (**a**) isothermal calorimetry, (**b**) total heat evolution curves, and (**c**) cement diffraction pattern.

The cement used in the composition of concrete mixes met the criteria of reduced alkali content and the specific surface area (see Figure 3). The identified crystalline components are clinker phases (alite -C_3_S, belite -C_2_S, C_4_AF, C_3_A, traces of CaO and MgO), and setting time regulators (gypsum, anhydrite, traces of bassanite) and carbonation products (calcite). 

### 3.2. Materials: Ceramic Dust

**Figure 4:**
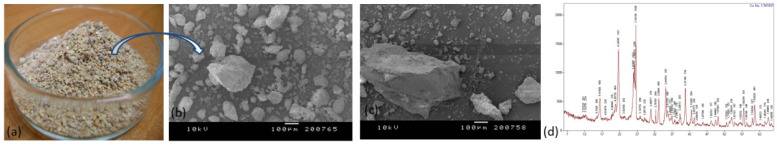
The ceramic dust intended for the laboratory tests: (**a**) natural sample, (**b**,**c**) sample observed in SEM, (**d**) diffractogram of the sample composition.

Ceramic dust included crystalline components: quartz, mullite, cristobalite, corundum, very small amounts of clay minerals, and anorthite [81]. The characteristics of the ceramic dust are presented in Figure 4.

**Figure 5:**
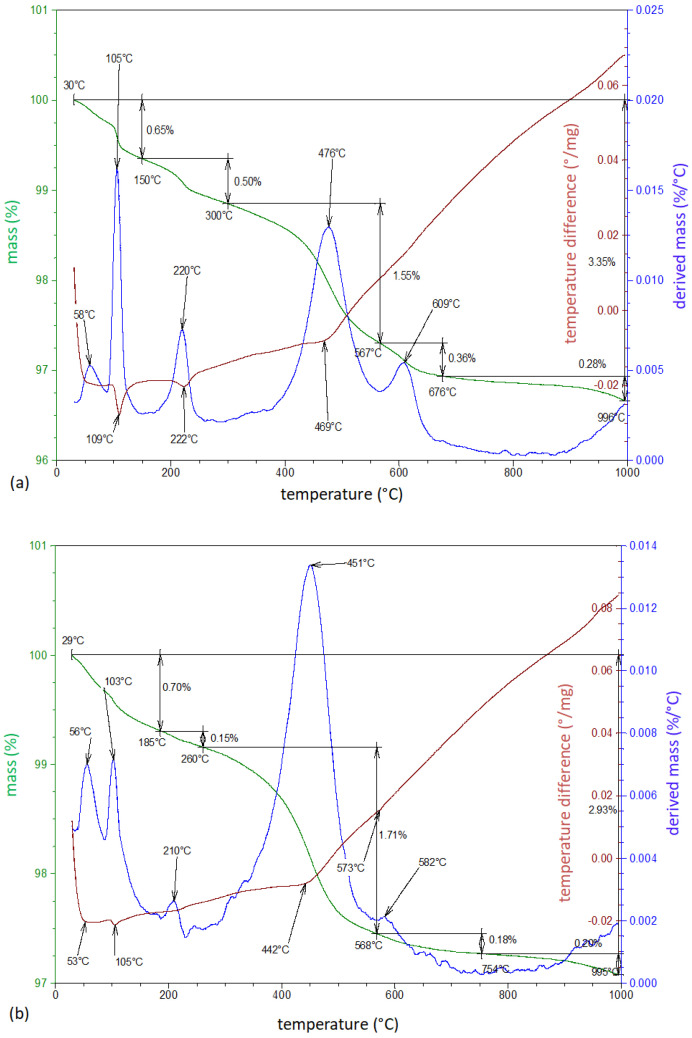
Thermogram sample of the ceramic dust with a fraction of 0/1 mm (**a**) and 0/2 mm (**b**).

Based on the results obtained from the thermal analysis of the ceramic dust used (Figure 5), it was found that in the case of dust with grain size 0/1 mm (a) in the temperature range of 20–150 °C and for dust with grain size 0/2 mm (b) in the temperature range of 20–185 °C, there is free and bound water in the relics of hydrated minerals clayey. Transformation of kaolinite to metakaolinite occurs in the temperature range of 300–567 °C (for dust with grain size 0/1 mm) and in the temperature range of 200–568 °C (for dust with grain size 0/2 mm). The decomposition of carbonate minerals occurs in the case of dust of 0/1 mm fraction in the temperature range 567 °C–676 °C, and for the dust of 0/2 mm fraction in the temperature range of 568 °C–754 °C. The content of carbonate minerals in terms of calcite is 0.8% and 0.4%, respectively. Above the temperature of 676 °C and 754 °C, the decomposition of metakaolinite was found in the dust with a grain size of 0/1 mm and 0/2 mm. The losses on roasting amounted to 3.35% for the dust of 0/1 mm and 2.93% for the dust of 0/2 mm.

The analysis of the research results to date, especially the parameters of the proposed ceramic dust (high strength and resistance to changing thermal conditions), indicates the possibility of applying an additive to the composition of airport concrete. Particularly beneficial is the effect of ceramic dust, as noted in [41,81], on changes in the porosity structure and internal structure of the concrete composite. It was found that the favorable changes are manifested in the range of hydration products that appear and the size of individual crystals. The proposed mix also reduces the shrinkage strains generated in concrete, both in the case of concrete maturing under standard conditions and concrete subjected to alternating thermal cycles. The dust suggested for use reduces the susceptibility of the cement matrix to the appearance of microcracks, also in the contact zones, which translates into higher strength values obtained. The beneficial effect is also magnified by the reduction of the amount of ettringite crystals, which have the possibility of growth and destruction of the surrounding zones. The change in the porosity characteristics in concrete (reduction in the diameter of air pores and the distance between them) resulting from the use of ceramic dust in combination with a smaller number of microcracks and a dense internal structure translates into a significant reduction in the water absorption of this concrete. This property of concrete is also visible in concrete exposed to the effects of thermal cycles.

### 3.3. Concrete Mix Design

At the mix design stage, preliminary assumptions were accepted, which included defining the following:

- assumed exposure classes (according to [59]) reflecting the influence of real environmental conditions on construction concrete—XM1, XC4, XA2, XM3, and XF4;
- assumed concrete strength class (according to provisions of [59])—C40/50;
- W/C mix coefficient—indicating the quality of slurry, each time this value did not exceed the limit value of 0,4, which was assumed according to standard requirements of [56], as the maximum value in case of mix designed for airfield pavements. Increased w/c coefficient, according to [18], is reflected in the increased porosity, water, and air permeability, increased volume changes during drying out, and at the same time decreasing the strength and resistance to environmental impact;
- minimum cement content in the concrete mix, as derived from the assumed concrete exposure class, which should not be lower than that presented in Table 1;
- total cement and fine aggregate content at a maximum level up to 450 kg/m^3^;
- required air content in the concrete mix—each time the air content w defined in accordance with [83] and was within the range from 4.5% to 5.5%, which was accepted according to standard requirements of [56], was the recommended value in case of mixes intended for airfield pavements;
- grain size distribution curve of concrete mix—each of the designed curves in case of concrete mixes was within good grain size distribution, accepted according to the requirements of [56] depending on the assumed method of laying the mixes within the structure.

According to our own research, the aggregate composition of concrete mix was diverse in individual components content and complied with aggregate composition tightness requirements. In the case of each mix type, the coarse aggregate was considered the basis. Fine aggregate in the form of washed sand was also taken into consideration in aggregate composition. Considering the analyzed research problem, there is a quantitative diversification of individual mix components. 

From the designing perspective of cement concrete intended for airfield pavements, the significant factor was the selection of coarse aggregate used in the case of aggregate composition. The designing process of specific mixes was planned according to analytical and experimental methods taking into consideration the following, which defines the necessary amount of individual components based on three equation methods according to [23]; verification of the obtained test results of mixtures testing with regard to consistency with the preliminary assumptions; experimental selection of mix composition. 

**Figure 6:**
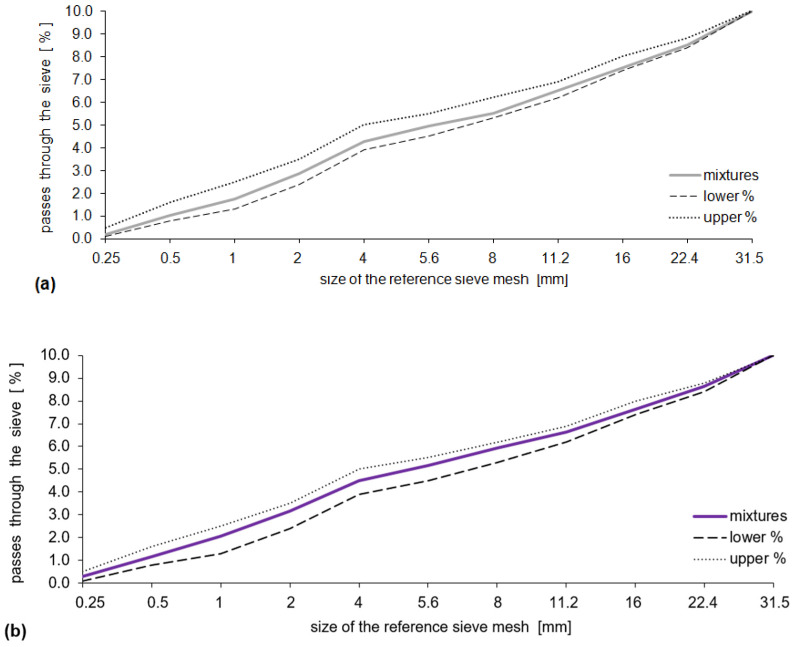
Aggregate compositions of CC-1 (**a**) and CC-2 (**b**) mixes.

The designed aggregate mixtures (Table 5), which were discussed in detail in the works [2,81], fit the range of good particle size distribution specified in the requirements [56] (see Figure 6). For the purposes of this study, the analyses included two types of concretes, without (CC-1) and with the proposed addition of ceramic flour (CC-2). In both analyzed compositions of concrete mixes, compatible chemical admixtures (air-entraining and plasticizing) were included.

### 3.4. Research Methodology

**Figure 7:**
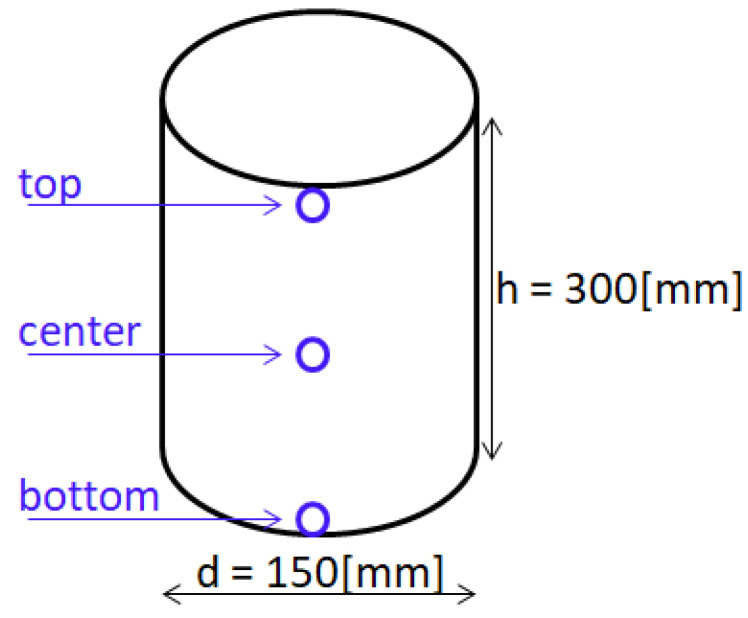
Distribution of laboratory test sites for samples in scheme A.

Concrete samples of CC-1 and CC-2 series, after 28 days of curing in accordance with the requirements of the [56] standard, were designated for experimental tests. The research consisted of determining the influence of the temperature generated from the aircraft engine operated in Poland. For the purposes of the experiment, a test stand was made that allowed directing the flue gas stream with strictly defined characteristics onto the samples according to the guidelines described in [81]. The tests were carried out for two diagrams: A and B. The first scheme—A—included the continuous impact of the flue gas stream on the samples for a period of 350 min with a test step every 25 min. The duration of annealing was selected for a comparative purpose, allowing the verification of the exposure time in scheme B. Comparative and proper samples (three in each series) were tested and stored at a reduced temperature (5 ± 2 °C). The comparative samples were stored under laboratory conditions. The proper samples were subjected to the annealing process. During measurements on each sample in selected places (see Figure 7), automatic temperature measurements were performed. The second diagram—B—reflected the real load on the airport rigid pavement in Poland. It was assumed that the samples were alternately heated and cooled in a time simulating successive take-offs at the airport. A single test cycle was modeled for a period of 16 min. The research period included 15 min of sample cooling in laboratory conditions and the impact of the stream of exhaust gases for 1 min. The number of 350 research cycles was adopted for further analysis. Samples stored at a temperature of 20 ± 2 °C were subjected to testing.

The arithmetic mean was used as a position measure [73] according to the Equation (1):

(1) $$\bar{x}=\frac{1}{n}\;\overset{n}{\underset{i=1}{\sum }}x_{i}$$

where: *n_i_* is the number of events in the group and *x_i_* is the successive values of the variable.

Standard deviation and a relative measure of dispersion (coefficient of variation to compare the measurements of the variation of the two characteristics or traits in different groups) were determined from Equations (2) and (3) according to [84].

(2) $$SD=\sqrt{\frac{1}{n}\;\overset{n}{\underset{i=1}{\sum }}{\left(x_{i}-\bar{x}\right)}^{2}}$$

(3) $$V=\frac{SD}{\bar{x}}\;\cdot \;100\%$$

The analyzes were performed with the Student’s *t*-test (Equation (4) according to [84]) for unrelated variables. The minimum number of samples varied between 4 and 12 depending on the type of the conducted research and taking into consideration the standard requirements. It was assumed that the compared groups of CC-1 and CC-2 concrete were of the same size and the distribution of results in each of the analyzed groups were comparable with the normal distribution. It was also assumed that the variances were similar and the dependent variable was expressed on a quantitative scale

(4) $$t=\frac{\bar{x_{1}}-\bar{x_{2}}}{\sqrt{\frac{SD_{1}^{2}\;\left(n_{1}-1\right)+SD_{1}^{2}\;\left(n_{2}-1\right)}{n_{1}+n_{2}-2}\;\left(\frac{1}{n_{1}}+\frac{1}{n_{2}}\right)}}$$

where: *n_i_* is the size of the *i*-th group. $\bar{x_{i}}$ is the mean of the *i*-th group, *SD_i_* is thestandard deviation of the *i*-th group.

Determining the compressive strength was conducted in cubic samples of the following dimensions 15 × 15 × 15 cm, according to the requirements of [85]. Every sample was analyzed in terms of its compliance with the declared dimensions and acceptable deviations [85]. Only samples that complied with the declared dimensions (<±0.5%—dimensional tolerance between surfaces obtained in the form, <±1.0%—dimensional tolerance between the upper smoothed surface and lower one obtained from the form, <±0.0006d—surface flatness tolerance on which the loading is transferred, <0.5 mm—perpendicularity tolerance of the side planes of the cube with regards to cube base) were intended for the compressive strength evaluation. Destructive tests were conducted in an automatic concrete press, in compliance with [86]. The test method included placing the sample in the central area of the press with regard to perpendicular loading surfaces to the direction of forming thereof. The samples were loaded with the constant speed of 0.5 MPa/s, which meets the requirements of [74] being within the range from 0.2 to 1.0 MPa/s. Only the results obtained for samples where the destruction process proved to be a properly conducted test according to [74] were taken into consideration for further analyses. Based on the maximum load value upon destruction (*F*) referred to sample cross-sectional area (*A_c_*), to which the destructive force is applied, the compressive strength was defined (*f_c_*), with the value approximately equal to 0.5 MPa—Equation (5). In the case of each concrete series, basic statistical characteristics were defined which allowed concrete verification in terms of its compliance with the standard requirements of [75].

(5) $$f_{c}=\frac{F}{A_{c}}$$

The qualitative phase composition tests were carried out in a TUR-M62 diffractometer (radiation—CuKα, filter—monochromator, lamp current-voltage—40 kV, lamp current intensity—20 mA, step—0.05 s, time constant—5 s). The quantification of selected components was performed on a thermal analysis device (DTG, DTA, and TG) of the SDT Q600 type (furnace atmosphere—air, heating rate—10 °/min, platinum crucibles).

Selected samples in the case of each concrete series after the assumed curing periods were intended for microscope observation in the scanning electron microscope and computed tomography. Microscope analysis required earlier preparation of concrete samples according to procedures and methodology of individual microscope techniques.

Microscope analysis was conducted by means of scanning electron microscope Zeiss-SUPRA type and FEI Quanta 250 FEG. Recent fractures or micro sections were prepared each time from concrete samples. In order to eliminate the negative influence of electron beam on the image and therefore improve the quality, each sample was sprayed with alayer of platinum 10 nm thick in the device Baltec SCD 005 attachment CEA 035. The surface of preparations was subject to observations by means of SEM and it was smaller than 1.0 cm^2^. Preparation of samples and interpretation of the obtained results were in compliance with those described in literature works [34,87]. The range of the applied magnifications ranged from 200× to 100,000×. Ref. [41] Using the scanning electron microscope allowed the assessment of changes occurring in the hardened concrete structure as a result of modification of the concrete mix composition. Single observations proved changes in the amount and morphology of the occurred crystals, size and components orientation as well as chemical composition and porosity characteristics. 

Examination of the analyzed concrete samples was conducted by means of computer tomography V/Tome made by GE, as described in the work [88]. The examined objects (samples of the following dimensions 150 × 150 × 150 mm) were placed on the rotating table and lamps (of 300 kV and 180 kV) and the detector remained immovable. The image of the examined samples was obtained after a full rotation was made. In the case of TEM microscopes, the electron stream “passes through” the prepared sample. Part of the electrons was absorbed or reflected, and the remaining part passed through the sample and was registered, which allowed the creation of a two-dimensional image. Using computed tomography allowed us to obtain complete and laminar images of the examined cement concrete samples. Moreover, it was possible to measure precisely the individual elements of the internal concrete structure in all directions and locate possible damages or discontinuities.

## 4. Results and Discussion

### 4.1. Diagram A

**Figure 8:**
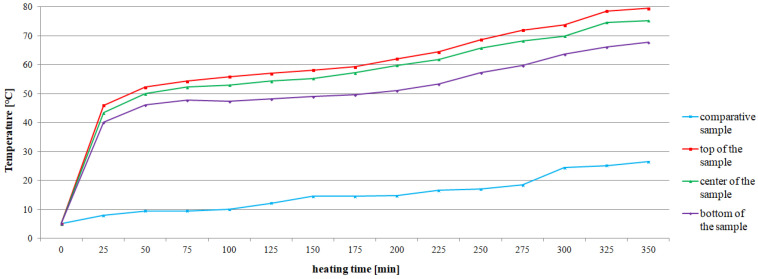
Change of the average temperature of CC-1 concrete as a function of heating time and test site on the sample.

**Figure 9:**
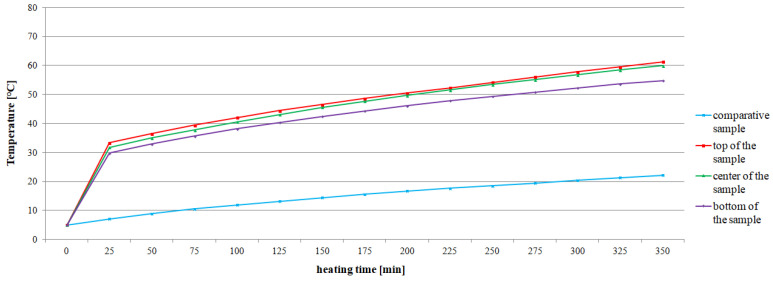
Change of the average temperature of CC-2 concrete as a function of heating time and test site on the sample.

The average values of the recorded temperatures are shown in Figure 8 and Figure 9. The comparative samples of concrete CC-1 and CC-2 were characterized by the lowest average temperature, changing respectively in the range from 5 °C to 26.5 °C and from 5 °C to 22.3 °C. In the case of the actual samples, an increase in temperature due to the action of exhaust gases was found, regardless of the test site. The average temperature values for CC-1 concrete (Figure 8) increased by 27.7 °C in the range from 40.1 °C to 67.8 °C for the bottom of the sample. For the center of the sample, the growth was 31.9 °C and the temperature ranged from 43.4 °C to 75.3 °C. For the top of the sample, the temperature difference was 33.5 °C from 46 °C to 79.5 °C. On the other hand, for CC-2 concrete, the recorded temperature increase was more stable and fluctuated within the range of 25–28 °C (Figure 9). In the case of the bottom of the sample, the temperature difference was 25.0 °C and ranged from 29.8 °C to 54.8 °C. For the center of the sample, the difference was 28.1 °C with a range from 31.8 °C to 59.9 °C. For the top of the sample, the difference was 28.0 °C and ranged from 33.3 °C to 61.3 °C. 

The temperature increase recorded for each successive 25 min of heating is summarized in Table 6. It was found that the temperature increase for CC-1 concrete ranged from 0.9 °C to 6.3 °C for the top of the sample, from 0.7 °C to 6.6 °C for the center of the sample, and from 0.3 °C to 6.0 °C for the bottom of the sample. However, the temperature increase for CC-2 concrete ranged from 1.7 °C to 3.2 °C for the top of the sample, from 1.5 °C to 3.2 °C for the center of the sample, and from 1.2 °C to 3.1 °C for the bottom of the sample.

It was found that the temperature change of CC-1 concrete was more heterogeneous than that of CC-2 concrete (Table 7). The recorded temperature changes of the samples after each subsequent 25 min for CC-1 concrete ranged from 2 °C to 4.2 °C for the top-center zone of the sample, from 3.3 °C to 8.8 °C for the center-bottom of the sample zones, and 5.9 °C to 12.7 °C for the top-bottom of the sample zone. However, for CC-2 concrete, they were respectively from 0.7 °C to 1.6 °C for the top-center of the sample zone, from 2.0 °C to 5.1 °C for the center-bottom of the sample zone, and 3.5 °C to 6.5 °C for the top-bottom of the sample zone. 

**Figure 10:**
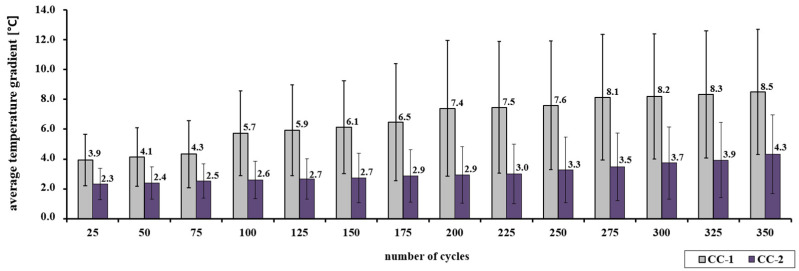
Gradient of the average temperature of the CC-1 and CC-2 concrete depending on the number of thermal cycles.

Statistical analysis showed that for individual test series the values obtained by CC-1 and CC-2 concrete are clearly different (see Table 7). For example, for a series of concretes heated for 350 min, the average value of the CC-1 concrete temperature gradient is 7.8 °C with a deviation of 3.8 °C, while for CC-2 concrete the average value is 4.3 °C with a deviation of 2.6 °C. The differentiation of the mean values for the CC-1 ranges from 3.9 °C to 8.5 °C. However, for CC-2 concrete, the values range from 2.3 °C to 4.3 °C. The recorded temperature rise for CC-2 concrete is lower and more stable as shown in Figure 10.

### 4.2. Diagram B

**Figure 11:**
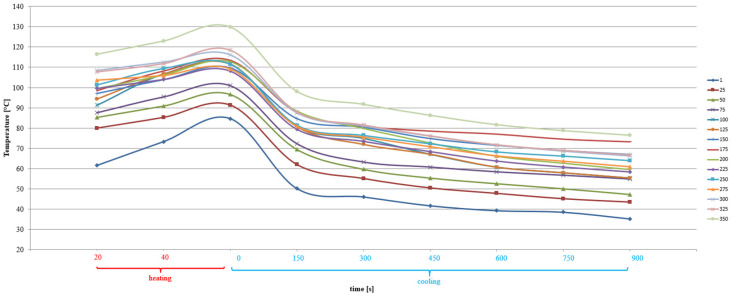
Change of the average temperature of CC-1 concrete as a function of heating and cooling time after a varied number of thermal cycles.

**Figure 12:**
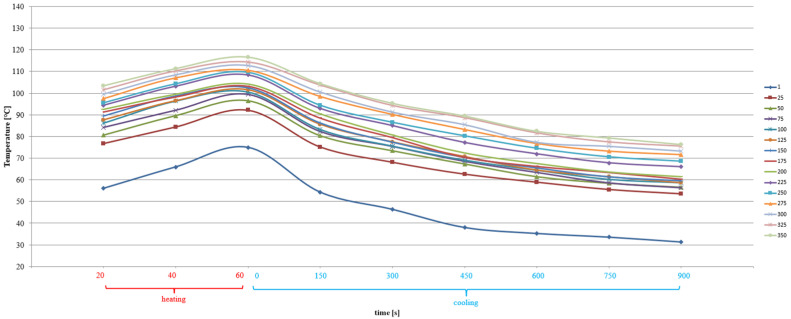
Change of the average temperature of CC-2 concrete as a function of heating and cooling time after a varied number of thermal cycles.

CC-1 and CC-2 concrete, exposed to alternating cycles of heating and cooling, changed their thermal parameters (Figure 11 and Figure 12). The average concrete temperature of CC-1 at the annealing stage increased from 61.5 °C to 129.8 °C after 350 cycles, while in the cooling stage after the maximum number of cycles it dropped to 76.4 °C. In the case of CC-2 concrete at the annealing stage, the temperature increase ranged from 56.1 °C to 116.6 °C after 350 cycles, while the temperature in the cooling stage dropped to 76.3 °C after the maximum number of cycles.

**Figure 13:**
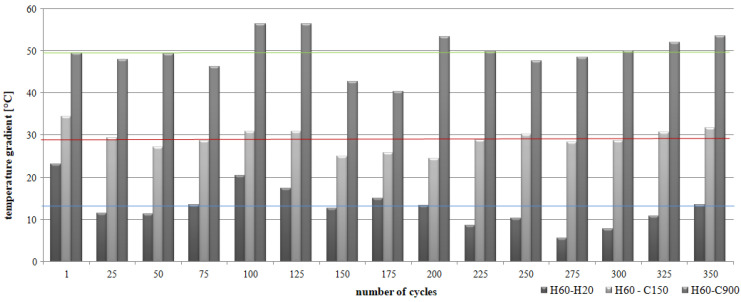
Gradient of the average temperature of the CC-1 concrete depending on the number of thermal cycles and the research period.

In the case of alternating heating cycles, a significant differentiation of the temperature gradient in individual periods was found for CC-1 and CC-2 concrete. For CC-1 concrete (Figure 13), the highest values of temperature differences were recorded by comparing the average temperatures after 60 s of annealing and 900 s of cooling of the concrete (H60-C900). The registered differences ranged from 40.3 °C to 56.3 °C, with the mean value at the level of 49.5 °C and the deviation of 4.47 °C. In the case of analyses of average temperatures after 60 s of concrete heating and 150 s of cooling (H60-C150), the differences ranged from 24.3 °C to 34.4 °C, with an average value of 28.9 °C and a deviation of 2.67 °C. The lowest temperature differences were recorded by comparing the mean values after 60 s of soaking (H60-H20). The recorded differences ranged from 5.5 °C to 23.1 °C, with the mean value at the level of 12.9 °C and a deviation of 4.62 °C.

**Figure 14:**
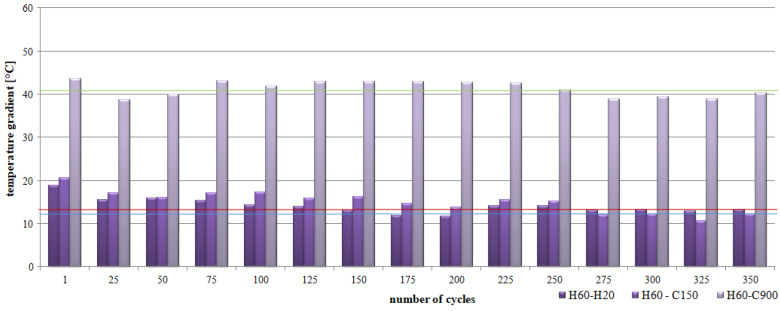
Gradient of the average temperature of the CC-2 concrete depending on the number of thermal cycles and the research period.

Additionally, for CC-2 concrete (Figure 14), the highest values of temperature differences were recorded by comparing the average temperatures after 60 s of heating and 900 s of cooling the concrete (H60-C900). The recorded differences were significantly lower than for CC-1 concrete and ranged from 38.7 °C to 43.5 °C, with an average value of 41.3 °C and a deviation of 1.78 °C. In the case of analyses of average temperatures after 60 s of heating and 150 s of cooling down of the concrete (H60-C150), the differences ranged from 10.6 °C to 20.5 °C, with an average value of 15.1 °C and a deviation of 2, 59 °C. Comparable temperature differences were recorded by comparing the mean values after 60 s of soaking (H60-H20). The recorded differences ranged from 11.7 °C to 18.8 °C, with the mean value at the level of 14.0 °C and the deviation of 1.79 °C.

**Figure 15:**
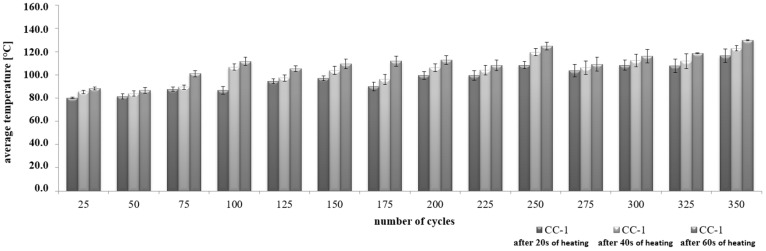
Change in the average temperature of CC-1 concrete during the heating process.

**Figure 16:**
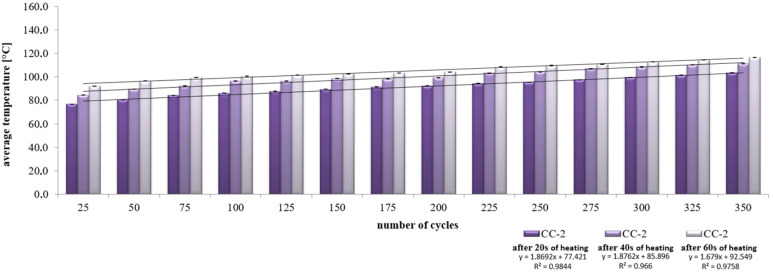
Change in the average temperature of CC-2 concrete during the heating process.

It was found that at the annealing stage, the increase in the average concrete temperature for CC-1 and CC-2 concrete varied, as shown in Figure 15 and Figure 16. The values ranged from 80 °C after 25 thermal cycles to 116.4 °C after 350 cycles. After 40 s of heating, the mean values ranged from 83.8 °C to 122.9 °C after 350 cycles, respectively. On the other hand, after 60 s of heating, the mean temperature values ranged from 88.4 °C to 129.8 °C. Temperature changes were subject to abrupt fluctuations.

In the case of CC-2 concrete, after 20 s of heating, the average values ranged from 76.7 °C after 25 thermal cycles to 103.4 °C after 350 cycles. After 40 s of soaking, the mean values ranged from 84.4 °C to 111.3 °C after 350 cycles, respectively. On the other hand, after 60 s of heating, the mean temperature values ranged from 92.2 °C to 116.6 °C. Temperature changes were always characterized by a linear course.

**Figure 17:**
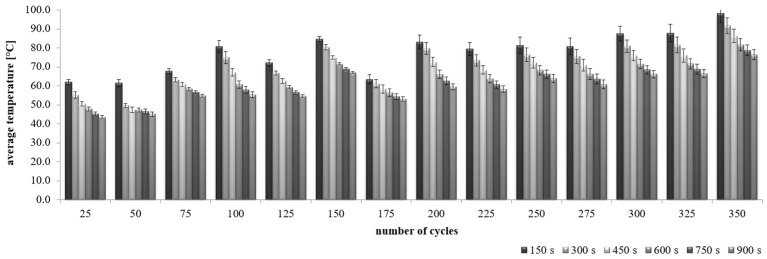
Change in the average temperature of CC-1 concrete during the cooling process.

**Figure 18:**
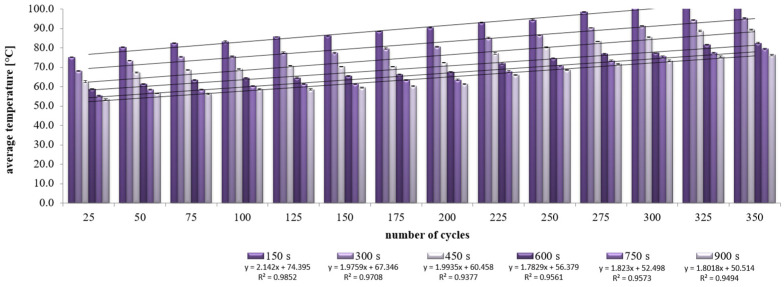
Change in the average temperature of CC-2 concrete during the cooling process.

The nature of sample cooling after the annealing process is different for CC-1 and CC-2 concrete (Figure 17 and Figure 18). In the first research period (after 150 s), the recorded mean temperature differences were the highest in both cases. For CC-1 concrete, the differences ranged from 61.5 °C (SD = 1.3 °C) to 98.1 °C (SD = 4.9 °C), and for CC-2 concrete it ranged from 75.2 °C (SD = 0.1 °C) to 104.4 °C (SD = 0.4 °C). These changes were a consequence of the end of the heating process. In the subsequent periods, when the concretes were continuously cooled, successive changes in average temperatures ($\bar{\chi }$) were lower in the case of CC-2 concrete. At the same time, greater differentiation of the recorded changes in the average temperature of CC-1 concrete was found after subsequent research periods (ss Table 8). On the basis of the obtained results, it was found that the CC-1 concrete is characterized by greater differentiation in relation to the average value in all analyzed periods.

### 4.3. Compressive Strength

The analysis of the change in the mean value of compressive strength was carried out for the concretes in diagram A and diagram B.

**Figure 19:**
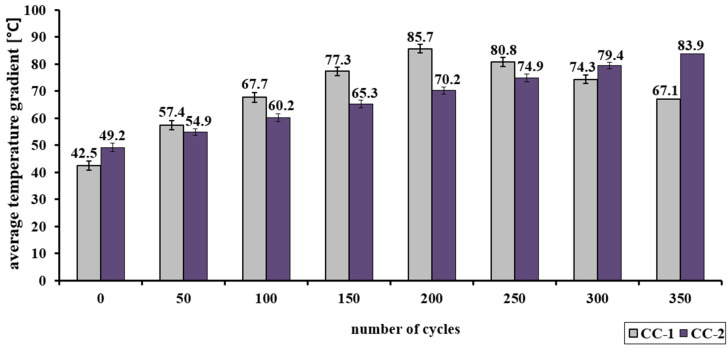
Change of the average compressive strength of CC-1 and CC-2 concrete as a function of the number of cycles—scheme A.

The analysis of changes in the average compressive strength of concrete CC-1 and CC-2 (diagram A) showed a significant differentiation (Table 9 and Figure 19). In the case of CC-1 concrete from 0 to 200 annealing cycles, the average value of the strength increases to nearly 86 MPa Concrete CC-2 subjected to 350 heating cycles is characterized by an increase in strength, to the value of nearly 84 MPa.

**Figure 20:**
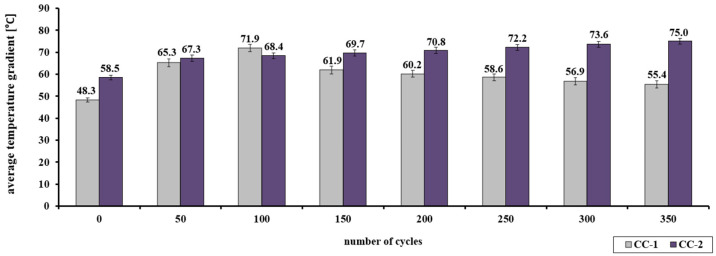
Change of the average compressive strength of CC-1 and CC-2 concrete as a function of the number of cycles—scheme B.

The analysis of changes in the average compressive strength of concrete CC-1 and CC-2 (diagram B) showed a significant differentiation (Table 10 and Figure 20). In the case of CC-1 concrete, from 0 to 100 annealing cycles, the average value of the strength increases to nearly 72 MPa. On the other hand, above 100 annealing cycles, a decrease in the examined feature is registered. Concrete CC-2 subjected to 350 heating cycles is characterized by an increase in strength, to the value of over 75 MPa. 

### 4.4. Internal Structure of Cement Concretes

**Figure 21:**
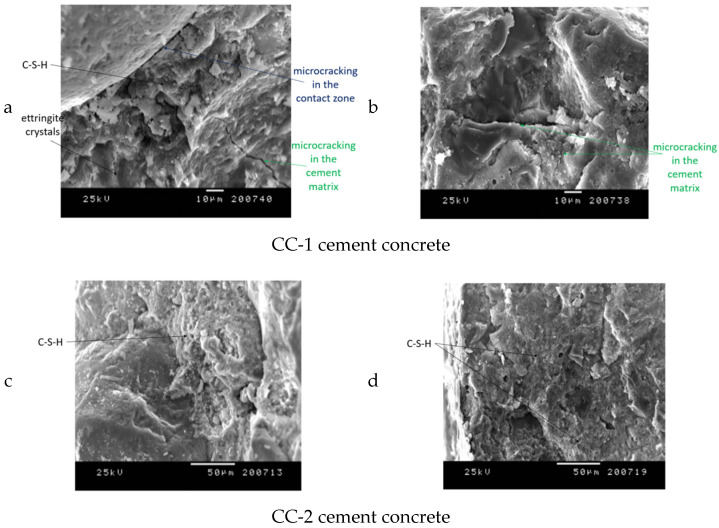
Construction of the internal structure of concrete subjected to thermal cycles according to scheme A for CC-1 and CC-2 concrete: (**a**,**c**) contact zone between the cement matrix and the aggregate grain, (**b**,**d**) cement matrix.

**Figure 22:**
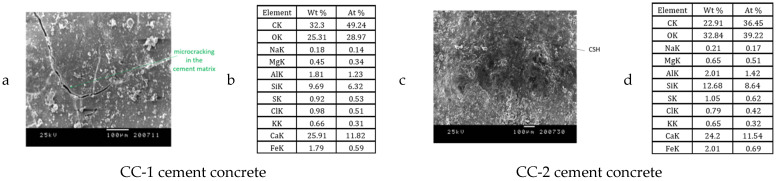
Concrete surface subjected to thermal cycles according to scheme A for CC-1 and CC-2 concrete: (**a**,**c**) sample surface, (**b**,**d**) chemical microanalysis.

**Figure 23:**
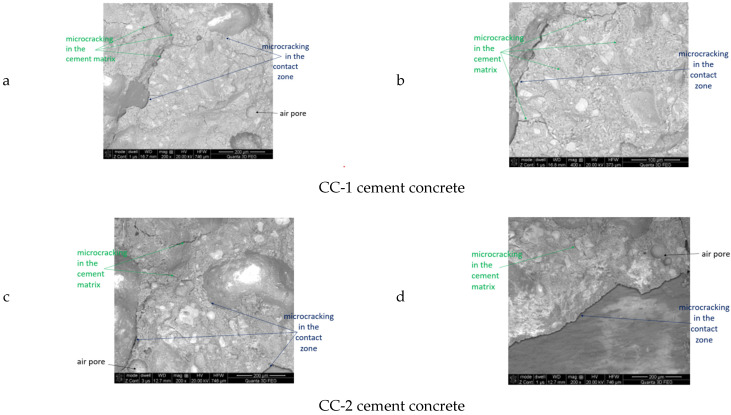
Construction of the internal structure of concrete subjected to thermal cycles according to scheme B for CC-1 and CC-2 concrete: (**a**,**c**) cement matrix, (**b**,**d**) contact zone between the cement matrix and the aggregate grain.

**Figure 24:**
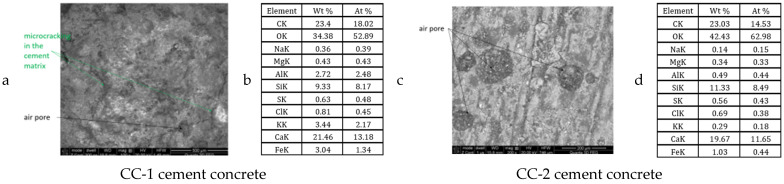
Concrete subjected to thermal cycles according to scheme B for CC-1 and CC-2 concrete: (**a**,**c**) sample surface, (**b**,**d**) chemical microanalysis.

Exemplary microphotographs obtained during SEM observations are presented in Figure 21, Figure 22, Figure 23 and Figure 24. 

The results observed in [70] allowed us to state that the CC-2 concrete analyzed after 28 days of maturation, is distinguished by a compact microstructure without visible microcracks. In the case of CC-1 concrete, micro-cracks of width up to 6 µm in cement matrix were proved after 28 days of curing and up to 2 µm in contact areas between aggregate grains and matrix. The differentiation of the internal structure of CC-2 concrete in relation to CC-1 concrete also applies to the reduction of the occurrence of ettringite crystals. The ettringite crystals in the CC-1 concrete matrix were characterized by large accumulation and crystal length. The CC-2 concrete was dominated by the crystallization of hydrated calcium silicates, which were in the form of a plate.

It was found that the CC-1 and CC-2 concrete samples, which were subjected to thermal cycles according to scheme A, were clearly differentiated in terms of internal structure (Figure 21 and Figure 22). In the case of concrete of the CC-1 series, crystallization of portlandite in the form of plates with a maximum size of up to 10 µm occurred. Concentrations of ettringite crystals with a length of up to 8 µm were also found in the cement matrix. Crystallization of hydrated calcium silicates of C-S-H type occurred in plate-granular form. In the case of concrete of series CC-2, a different crystallization was demonstrated. There were smaller amounts of portlandite crystals and ettringite crystals. The size of a single ettringite crystal did not exceed 5 µm. The differences also concerned the structure of the contact surface of the aggregate grain with the cement matrix. The contact surface of the matrix with aggregate in the case of CC-1 concrete consisted of ettringite crystals up to 5 μm long. The contact surface of the aggregate grains with the cement matrix in the case of CC-2 concrete consisted of fine-fiber C-S-H and a much smaller proportion of ettringite crystals. The crystal size did not exceed 1 μn. The contact surfaces of the cement matrix and the grains of the ceramic modifier in the case of the CC-2 series concrete were continuous, there were no microcracks even after the maximum number of thermal cycles. In the case of the surface zone, directly exposed to thermal cycles, microcracks occurred in CC-1 concrete and CC-2 concrete. In the case of CC-2 concrete, they were much less developed and did not exceed 4 μm. In the case of CC-1 concrete, the microcracks were up to 14 μm wide.

It was found that the CC-1 and CC-2 concrete samples, which were subjected to thermal cycles according to scheme B, were clearly differentiated in terms of internal structure (Figure 23 and Figure 24). In the case of concrete of the CC-1 series, crystallization of portlandite in the form of plates with a maximum size of up to 10 µm occurred. Concentrations of ettringite crystals with a length of up to 10 µm were also found in the cement matrix. Crystallization of hydrated calcium silicates of C-S-H type occurred in granular form. In the case of concrete of series CC-2, a different crystallization was demonstrated. There were portlandite crystals (more elaborate) and smaller amounts of ettringite crystals. The size of a single C-S-H crystal did not exceed 7 µm. The differences also concerned the structure of the contact surface of the aggregate grain with the cement matrix. The contact surface of the matrix with aggregate in the case of CC-1 concrete consisted of ettringite crystals up to 8 μm long. In these zones, microcracks were observed, which resulted in a reduction in mechanical parameters. The contact surface of the aggregate grains with the cement matrix in the case of CC-2 concrete consisted of fine-fiber C-S-H and a much smaller proportion of ettringite crystals. The contact surfaces of the cement matrix and the grains of the ceramic modifier in the case of the CC-2 series concrete were continuous, there were no microcracks even after the maximum number of thermal cycles. In the case of the surface zone, directly exposed to thermal cycles, microcracks occurred in CC-1 concrete and CC-2 concrete. In the case of CC-2 concrete, they were much less developed and did not exceed 5 μm. In the case of concrete, CC-1 was up to 35 μm wide.

**Figure 25:**
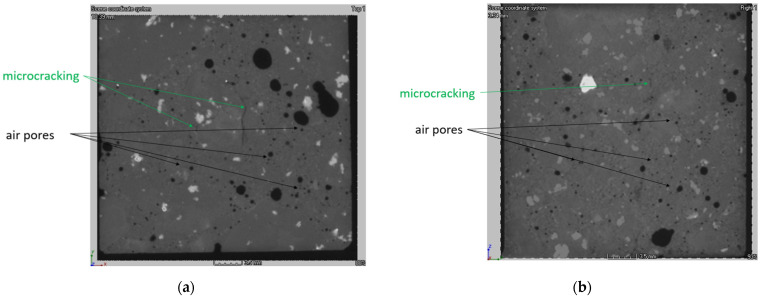
This is a figure. Schemes follow another format. If there are multiple panels, they should be listed as: The internal structure of concrete series CC-1 (**a**) and series CC-2 (**b**).

Exemplary data obtained during the TEM observations are presented in Figure 25. The relationships observed for concretes in the SEM observations were confirmed by the observations in TEM. It was found that there are significant differences in the volume of the analyzed CC-1 and CC-2 concrete samples. The overall content of porosity in CC-1 concrete is lower, and the pores present are characterized by larger diameters. In addition, the structure of the pore distribution in CC-2 concrete is more homogeneous (Table 11). 

The concrete compositions analyzed in the study differed in the material composition and the share of hydration products. This was the cause of heterogeneous stresses and the appearance of a distorted microstructure during linear changes during thermal cycles. It was observed that the ettringite in the heated CC-1 concrete was also present in the cracks, which proves the destruction of the microstructure. In each of the cases (Scheme A and Scheme B), the edge of the expanding cement slurry is a representation of the grain outline of the aggregate. This is due to the use of an aggregate that (as shown in the paper) does not expand. The changes in the volume of CC-1 concrete were caused by the expansion of the grout, which caused the microcracks to open in the contact zone and were the site of the development of ettringite crystals. In addition, ettringite crystals were developed. In the case of CC-2 concrete (after 28 days of maturation), there were no microcracks in the contact zone, which limited the propagation of cracks after thermal cycles and the aging process. The aging process in the case of concretes subjected to thermal diagrams consisted of the intensification of ettringite crystallization, especially in the existing microcracks and in the spaces of the contact zone between the aggregate grain and the cement matrix. The change in the length and geometry of the cracks is caused by the stresses appearing as a result of heating and cooling and is more intensively carried out according to the scheme in the B process. The CC-1 ettringite appearing in the microcracks in the concrete causes increased expansion and enlargement of the crack width. The appearance of microcracks in CC-1 concrete already after 28 days of maturation intensifies the range of crack opening after thermal cycles and, consequently, increases the expansion. The confirmation of the obtained results is consistent with the results presented in [88], which concerned increased contraction strains for this concrete. In the case of CC-2 concrete, the dense structure of the aggregate-matrix transition zone makes it difficult for the solution to move through the porous structure (contact zone) and thus slows down the formation of ettringite. The use of ceramic flour in this concrete, which is characterized by a very low coefficient of thermal expansion, also limited the expansion.

## 5. Conclusions

The presented research was carried out to assess the influence of thermal cycles on the change of selected physical and mechanical parameters and the structure of the internal microstructure of concrete intended for airport pavements. The laboratory results allowed for the formulation of the following conclusions:

1. Non-reactive aggregate and cement, which contains a reduced alkali content and ceramic dust in the concrete mix were used, produced concrete with increased resistance to the diversified influence of thermal cycles.

The increase in concrete resistance was registered regardless of the heating pattern. In the case of scheme A, after the impact of 350 thermal cycles, a higher increase in the average temperature of CC-1 concrete was observed than that of CC-2 concrete, in the range from 27.7 °C to 33.5 °C and in the range from 25 °C to 28 °C, respectively. The analysis of the temperature gradient in the 25-min interval showed that CC-2 concrete is characterized by lower values than CC-1 concrete, in the range from 1.2 °C to 3.2 °C and in the range from 0.7 °C to 6.6 °C, respectively. A change in the temperature gradient in terms of the sample cross-section was also observed. In this case, higher values were recorded for CC-1 concrete than for CC-2 concrete. The temperature gradient for CC-1 concrete in the top-bottom section of the sample was from 5.9 °C to 12.7 °C, in the top-center zone from 2.0 °C to 4.2 °C, and in the middle-bottom zone from 3.3 °C to 8.8 °C. In the case of CC-2 concrete, the temperature gradient in the top-bottom section of the sample was from 3.5 °C to 6.5 °C, in the top-center zone from 0.7 °C to 1.6 °C, and in the middle-bottom zone from 2.0 °C to 5.1 °C.

In the case of cyclic heating and cooling of concretes according to scheme B, it was found that CC-2 concrete heats up to lower temperatures than CC-1 concrete. The average temperature of CC-2 concrete was over 8 lower than that of CC-1 concrete. The temperature difference of CC-1 concrete analyzed after 60 s soaking and 900 s cooling was in the range from 40.3 °C to 56.3 °C, while for CC-2 concrete it was in the range from 38.7 °C to 43.5 °C, respectively. The temperature difference of CC-1 concrete analyzed after 60 s of heating and 150 s of cooling was in the range from 24.3 °C to 34.4 °C, while for CC-2 concrete it was in the range from 10.6 °C to 20.5 °C, respectively. The temperature difference of CC-1 concrete analyzed after 60 s heating and 20 s cooling down ranged from 5.5 °C to 23.1 °C, while for CC-2 concrete it was in the range from 11.7 °C to 18.8 °C, respectively.

2. Changes in the internal structure were observed, in particular concerning the porosity characteristics, the structure of the contact zone between the aggregate grain and the matrix, and the structure of the cement matrix.

In the case of annealing according to scheme A, it was observed that in the CC-2 concrete, less extensive ettringite crystallization occurred. It concerned the cement matrix and the contact zones between the matrix and aggregate grains. In the cement matrix, the length of individual crystals did not exceed 5 μm in CC-2 concrete, while in the CC-1 concrete it reached the value of 8 μm. There was also a noticeable difference in the contact zone between the cement matrix and aggregate grains, for CC-2 concrete the crystal length did not exceed 1 nm, and for CC-1 concrete it was 5 μm. The extensive crystallization of ettringite in CC-1 concrete increased the width of the microcracks. On the other hand, in CC-2 concrete, the contact zones between the aggregate grains and the cement matrix remained continuous. This change translated into the achievement of higher values of compressive strength of concrete subjected to annealing cycles.

In the case of annealing according to scheme B, it was observed that in the CC-2 concrete, less extensive ettringite crystallization occurred, in favor of extensive crystallization of portlandite. In the cement matrix of CC-1 concrete, the length of individual ettringite crystals was up to 10 μm. In the contact zone between the cement matrix and aggregate grains for CC-1 concrete, the length of the crystal reached 8 μm. On the other hand, in the CC-2 concrete, the contact zones between the aggregate grains and the cement matrix remained continuous, and the crystallization of hydrated calcium silicates with a size of up to 7 μm prevailed.

3. A significant increase in the compressive strength of concrete with the proposed ceramic dust was found, regardless of the course of the heating process.

In the case of CC-2 concrete, an increase in compressive strength was recorded in the range of up to 350 annealing cycles, regardless of the adopted scheme. In scheme A, the strength increased from 49.2 MPa to 83.9 MPa, and in scheme B, from 58.5 MPa to 75.0 MPa. However, in the case of concrete without the addition of the proposed dust (CC-1), the compressive strength in scheme A increased in the range from 42.5 MPa to 85.7 MPa after 200 cycles, and after subsequent cycles it decreased, reaching the average value of 67.1 MPa after 350 cycles. In scheme B, the tendency was similar, CC-1 concrete was characterized by an increase in strength up to 100 cycles (in the range from 48.3 MPa to 71.9 MPa), and then a decrease in strength by subsequent cycles to the value of 55.4 MPa after 350 cycles was observed.

## Figures and Tables

**Table 1:** Restrictions concerning the composition of concrete intended for airfield pavements.

Parameters	Environmental Impact				
	XA2	XC4	XD3	XF4	XM3
Minimum strength class	C30/37	C25/30	C35/45	C30/37	C35/45
Maximum water/cement ratio (w/c)	0.50	0.50	0.45	0.45	0.45
Minimum cement content CEM I 32.5 [kg/m^3^]	300	280	300	340	300
Minimum cement content CEM I 42.5 [kg/m^3^]	270	270	270	240	280

**Table 2:** Polish requirements for aggregates intended for airfield pavements.

Physical and Mechanical Properties	Aggregate Type	
	Coarse Aggregate	Fine Aggregate
Mineral fines content	≤1.0%	≤1.0%
Evenness index	≤15	-
Fragmentation resistance	≤40	-
Abrasion resistance	≤20%	-
polishing resistance	≥50	-
Grain absorbability	≤1.0%	-
Foreign impurities	≤0.1%	≤0.25%

**Table 3:** Physical properties of mixes.

Physical Properties		Method of Mix Incorporation	
		Slip Formwork Method	Slip Formwork Method
consistency	Determined by means of Vebe method acc. to [71]	≥31 s	20–30 s
	Determined by means of concrete slump test acc. to [72]	10.0–40.0 mm	50.0–90.0 mm
Air content determined by pressure methods acc. to [73]		4.5–5.5%	

**Table 4:** Properties of the applied aggregate.

Type of Aggregate	Granite Grit	Fine Aggregate
Shrinkage during drying [%]	<0.075	<0.025
Absorbability [%]	<1.0	<1.0

**Table 5:** Material composition of mixes of series CC-1 and CC-2.

Materials		Concrete CC-1	Concrete CC-2
Cement CEM I 42.5N		377	377
Granite grit	2/8 mm	670	670
	8/16 mm	160	160
	16/32 mm	570	570
Fine aggregate 0/2 mm		415	370
Ceramic dust 0/2 mm		0	45

**Table 6:** Gradient of the average temperature of the CC-1 and CC-2 concrete depending on the test site and heating time.

Sample	Heating Time [Minutes]													
	25	50	75	100	125	150	175	200	225	250	275	300	325	350
CC-1 concrete—temperature gradient [°C]														
Top	41	6.3	2	1.6	1.2	1.1	1.1	2.8	2.4	4.2	3.3	1.8	4.8	0.9
Center	38.4	6.6	2.3	0.7	1.3	0.9	2	2.6	2.1	3.9	2.4	1.7	4.7	0.7
Bottom	35.1	6	1.7	0.5	0.9	0.8	0.3	2.3	2.3	4	2.5	1.6	4.7	1.7
CC-2 concrete—temperature gradient [°C]														
Top	28.3	3.2	2.9	2.6	2.4	2.1	2.1	1.9	1.8	1.9	1.8	1.8	1.7	1.8
Center	26.8	3.2	2.8	2.8	2.5	2.5	2.1	2	1.9	1.8	1.7	1.7	1.6	1.5
Bottom	24.8	3.1	2.7	2.5	2.3	2	1.9	1.8	1.7	1.5	1.5	1.4	1.4	1.2

**Table 7:** Gradient of the average temperature of the CC-1 and CC-2 concrete depending on the heating time and zones.

CC-1 Concrete				CC-2 Concrete			
Heating Time [Minutes]	Temperature Gradient [°C]			Heating Time [Minutes]	Temperature Gradient [°C]		
	Top-Center	Center-Bottom	Top-Bottom		Top-Center	Center-Bottom	Top-Bottom
25	2.6	3.3	5.9	25	1.5	2	3.5
50	2.3	3.9	6.2	50	1.5	2.1	3.6
75	2.0	4.5	6.5	75	1.6	2.2	3.8
100	2.9	5.7	8.6	100	1.4	2.5	3.9
125	2.8	6.1	8.9	125	1.3	2.7	4
150	3.0	6.2	9.2	150	0.9	3.2	4.1
175	2.1	7.6	9.7	175	0.9	3.4	4.3
200	2.3	8.8	11.1	200	0.8	3.6	4.4
225	2.6	8.6	11.2	225	0.7	3.8	4.5
250	2.9	8.5	11.4	250	0.8	4.1	4.9
275	3.8	8.4	12.2	275	0.9	4.3	5.2
300	3.9	8.3	12.3	300	1	4.6	5.6
325	4.0	8.5	12.5	325	1.1	4.8	5.9
350	4.2	8.5	12.7	350	1.4	5.1	6.5

**Table 8:** Characteristics of the change in parameters of the average temperature of CC-1 and CC-2 concrete during the cooling process.

Value	Cooling Time [s]											
	150		300		450		600		750		900	
	Min	Max	Min	Max	Min	Max	Min	Max	Min	Max	Min	Max
CC-1 concrete—temperature gradient [°C]												
$\bar{\chi }$	61.5	98.1	49.7	91.7	47.4	86.3	47.4	81.6	45.2	78.7	43.5	76.4
SD	1.9	4.9	1.0	4.2	1.3	3.5	1.0	3.2	1.0	2.9	1.0	2.7
V	3.1	5.0	2.1	4.6	2.8	4.1	2.2	3.9	2.1	3.7	2.3	3.5
CC-2 concrete—temperature gradient [°C]												
$\bar{\chi }$	75.2	104.4	68.1	95.3	62.6	89.5	58.8	82.4	55.4	79.4	53.4	76.4
SD	0.1	0.3	0.2	0.3	0.5	0.3	0.2	0.4	0.3	0.3	0.3	0.4
V	0.1	0.3	0.3	0.3	0.8	0.3	0.4	0.4	0.5	0.4	0.6	0.5

**Table 9:** Characteristics of the change in the relative measure of the dispersion of the average compressive strength of concrete CC-1 and CC-2—scheme A.

Concrete	Number of Cycles							
	0	50	100	150	200	250	300	350
CC-1	2.73	3.35	2.54	2.35	1.97	2.36	2.52	2.56
CC-2	2.10	2.07	2.06	1.67	1.65	1.61	1.34	1.33

**Table 10:** Characteristics of the change in the relative measure of the dispersion of the average compressive strength of concrete CC-1 and CC-2—scheme B.

Concrete	Number of Cycles							
	0	50	100	150	200	250	300	350
CC-1	1.58	2.19	1.88	2.24	2.05	2.18	2.32	2.29
CC-2	1.12	1.77	1.54	1.55	1.57	1.46	1.49	1.32

**Table 11:** Concrete characteristics as specified in TEM.

Specific Characteristics of Concrete	Concrete					
	after 28 Days		after Scheme A		after Scheme B	
	CC-1	CC-2	CC-1	CC-2	CC-1	CC-2
test sample volume [mm^3^]	3,375,000	3,375,000	3,375,000	3,375,000	3,375,000	3,375,000
the void content of the entire sample [%]	3.81	4.36	4.15	4.51	4.32	4.62
total volume of voids [mm^3^]	37,896.46	42,162.19	4362.81	4295.48	4418.32	4315.46
the volume of the greatest voids [mm^3^]	1034.48	516.52	1560.75	630.79	1637.20	640.82
air pore content less than 300 μm in diameter [%]	0.23	0.34	0.19	0.32	0.17	0.31
volume of micropores [mm^3^]	3044.91	3824.71	2890.45	3750.39	2915.16	3790.30
round pore content [%]	0.11	0.19	0.10	0.18	0.10	0.18
volume of round pores [mm^3^]	351.83	602.15	343.65	586.12	325.20	572.31
